# Genetic differentiation underlies seasonal variation in thermal tolerance, body size, and plasticity in a short‐lived copepod

**DOI:** 10.1002/ece3.6851

**Published:** 2020-10-05

**Authors:** Matthew C. Sasaki, Hans G. Dam

**Affiliations:** ^1^ Department of Marine Sciences University of Connecticut Groton CT USA

**Keywords:** Copepod, fluctuating selection, phenotypic plasticity, seasonality, thermal tolerance, trade‐off

## Abstract

Organisms experience variation in the thermal environment on several different temporal scales, with seasonality being particularly prominent in temperate regions. For organisms with short generation times, seasonal variation is experienced across, rather than within, generations. How this affects the seasonal evolution of thermal tolerance and phenotypic plasticity is understudied, but has direct implications for the thermal ecology of these organisms. Here we document intra‐annual patterns of thermal tolerance in two species of *Acartia* copepods (Crustacea) from a highly seasonal estuary, showing strong variation across the annual temperature cycle. Common garden, split‐brood experiments indicate that this seasonal variation in thermal tolerance, along with seasonal variation in body size and phenotypic plasticity, is likely affected by genetic polymorphism. Our results show that adaptation to seasonal variation is important to consider when predicting how populations may respond to ongoing climate change.

## Graphical abstract

We examined seasonal variation in thermal performance curves of a short‐lived marine copepod. Experiments with unacclimated individuals from the field and laboratory common garden experiments indicate that the observed seasonal variation in both thermal tolerance and phenotypic plasticity likely has a genetic basis.

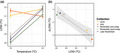

## 1 INTRODUCTION

Temperature varies in natural environments on several different time scales. Seasonality is an especially prominent form of variation in natural systems (Williams et al., 2017). Coastal waters and estuaries exhibit some of the most pronounced seasonality in temperature in marine environments. The evolutionary impacts of this seasonal variation are understudied, especially in organisms with short generation times relative to the annual temperature cycle. Identifying the adaptive mechanisms comprising adaptation to across‐generation seasonal variation in temperature strongly affects our ability to predict population responses to ongoing climate change in marine and terrestrial ecosystems.

Populations of short‐lived organisms can adapt to seasonality through several mechanisms, including genetic polymorphism and phenotypic plasticity. Populations often contain abundant adaptive genetic variation, which may promote rapid responses to changes in the environment (Bitter et al., 2019; Brennan et al., 2019). Fluctuating selection may result in stable oscillations in the relative abundance or frequency of different alleles in the population if they correspond to phenotypes adapted to different environments experienced throughout the year (a winter and a summer morph for example; Bergland et al., 2014). Phenotypic plasticity, the ability of a single genotype to produce multiple phenotypes in response to variation in some environmental cue, is also an important adaptive mechanism (Ghalambor et al., 2007; West‐Eberhard, 2003). By producing a relatively rapid improvement in the match between phenotype and environment, adaptive plasticity may promote population persistence in variable environments. The evolution of plasticity itself represents another adaptive mechanism that populations may use to cope with seasonality. The magnitude of the phenotypic response to a change in the environment, which we refer to as the strength of phenotypic plasticity, is heritable (Scheiner & Lyman, 1989, 1991) and may evolve in response to variability in the environment (Janzen, 1967; Stevens, 1989).

The effects of fluctuating or seasonally variable selection on genetic polymorphism and phenotypic plasticity have long been of interest in the study of evolutionary ecology (Ellner & Sasaki, 1996; Gilchrist, 1995; Haldane & Jayakar, 1963; Hoekstra, 1975). Several conditions and mechanisms promote the maintenance of genetic polymorphism by fluctuating selection, including sexual reproduction, overlapping generations, positive temporal autocorrelation, and seasonal changes in dominance (Ellner & Hairston, 1994; Ellner & Sasaki, 1996; Svardal et al., 2014; Tufto, 2015; Wittmann et al., 2017). Much of this work has focused on seasonal variation experienced within generations, rather than between generations, and the predictability and frequency of environmental variation relative to generation time is also important to consider. These characteristics determine how fluctuating selection affects not only the maintenance of genetic polymorphism, but also the evolution of phenotypic plasticity (Gilchrist, 1995; Levins, 1968; Wieczynski et al., 2018). Further, there are also likely interactions between the two adaptive mechanisms that affect the evolutionary dynamics of these systems. For example, there may be a trade‐off between tolerance and plasticity (Stillman, 2003), or phenotypic plasticity may dampen fluctuating selection and impede the maintenance of genetic polymorphism (Crispo, 2008; Ellner & Hairston, 1994).

There is abundant evidence for seasonally variable thermal tolerance in both vertebrate (Fangue & Bennett, 2003; Kowalski et al., 1978; Trullas & Chown, 2013) and invertebrate taxa (Berkelmans & Willis, 1999; Bujan et al., 2020; Hamdoun et al., 2003; Hopkin et al., 2006; Morley et al., 2012; Stickle et al., 2017). Much of this empirical literature focuses on long‐lived taxa which experience seasonality within a generation, rather than across generations. As such, seasonal variation in thermal tolerance likely represents the effects of phenotypic plasticity. While populations of short‐lived organisms may also display phenotypically plastic responses, variation in allele frequency can play a prominent role when seasonal variation occurs across generations (Bergland et al., 2014; Carvalho & Crisp, 1987; Dobzhansky, 1947; Dobzhansky & Ayala, 1973; Wormhoudt, 2015), particularly via the production of seasonal variation in thermal tolerance across generations (Bradley, 1978a, 1978b; Carvalho, 1987; Kenny et al., 2008; King, 1972). Seasonal variation in the strength of plasticity itself is much less well‐studied, but could also be important (Noh et al., 2017; Tsuji, 1988). Seasonal variation in plasticity may reflect fluctuating selection on plasticity itself by changes in the amount of within‐generation variation or could reflect the effects of a trade‐off between tolerance and plasticity (Stillman, 2003). Identifying the contributions of these adaptive mechanisms to observed variation in thermal tolerance can be challenging as it requires both the measurement of thermal tolerance on unacclimated individuals from the field, as well as laboratory common garden, split‐brood experiments to determine whether genetic differentiation affects either thermal tolerance or the strength of phenotypic plasticity.

Copepods are arguably the most abundant animals on Earth (Hardy, 1970; Humes, 1994; Huys & Boxs hall, 1991; Turner, 2004). Because they dominate zooplankton communities, they play crucial roles in both marine trophic webs and global biogeochemical cycles (Menden‐Deuer & Kiørboe, 2016). Understanding how this group may respond to ongoing climate change is crucial for predicting the future ecological dynamics in marine and freshwater systems (Dam, 2013). Many copepod species are broadly distributed and exhibit local thermal adaptation (Damgaard & Davenport, 1994; Kelly et al., 2011; Lonsdale & Levinton, 1985; Pereira et al., 2017; Sasaki & Dam, 2019). Many of these species also have ranges that extend into temperate coastal environments and therefore experience large degrees of seasonality within populations. As copepods often have short generation's times, any combination of the three discussed adaptive mechanisms (genetic polymorphism, phenotypic plasticity, and variation in the strength of plasticity) may play an important role in adaptation to seasonality. *Acartia* copepods are excellent model systems for studying these seasonal dynamics. Two Acartiid species dominate the planktonic community in Long Island Sound, a highly seasonal temperate estuary (annual water temperature range of ~25°C; Lopez et al., 2014). *Acartia hudsonica* is traditionally considered the winter dominant species, which is then replaced by *Acartia tonsa* as waters become warmer during the summer (Rice et al., 2014; Sullivan & McManus, 1986).

In this study, we show clear seasonal variation in thermal tolerance in the two species of *Acartia* copepods from Long Island Sound. Using split brood, common garden experiments, we then show that genetic differentiation between seasonal collections of the summer‐dominant species, *Acartia tonsa,* drives differences in thermal tolerance and body size, as well as in the strength of phenotypic plasticity of both traits. Understanding the seasonal dynamics of thermal adaptation and the mechanisms by which populations of copepods, and other short‐lived organisms, respond to this variability has significant implications for our understanding of the processes that generate and maintain adaptive variation in populations and for our ability to predict ecosystem dynamics in a changing climate.

## 2 METHODS

Copepods were collected in surface tows at irregular intervals from July 2017 to November 2019 from Eastern Long Island Sound (41.32 N, −72 W) on incoming tides using a 250‐µm mesh plankton net with a solid cod end (collections summarized in Appendix S1). Water depth at the collection site is ~1.5 m. Temperature and salinity at the surface were measured at the time of collection using a handheld thermometer and salinometer. Copepods were immediately taken to the University of Connecticut Avery Point Campus, where mature *Acartia* individuals were sorted into 0.2 µm filtered seawater and held at the temperature of collection. Collections were generally dominated by *Acartia tonsa* during Summer and Fall and *Acartia hudsonica* during late Winter, Spring, and early Summer, but there were several collections with the two species present in abundances high enough to warrant inclusion of both (Appendix S1). Individuals were allowed to rest for six hours at the temperature measured during collection to reduce stress associated with collection. After this resting period, individuals were exposed to an acute heat shock following protocols developed previously for Acartiid copepods (Sasaki & Dam, 2019; Sasaki et al., 2019). Briefly, mature females were gently transferred to a 2‐ml microfuge tube with 1.5 ml of filtered seawater. Tubes were partially capped to minimize evaporation, and therefore salinity fluctuations, while still allowing for gas exchange. Tubes were then placed into 15‐well dry heat baths (USA Scientific) which were set at a range of temperatures. Heat stress temperatures ranged between 10°C and 39°C, but differed between collections to cover the range of survivorship from 100% survival to 100% mortality. Each female experienced just one temperature during the heat stress and generally at least 12 females were used per temperature. After 24 hours, individuals were removed, and survivorship determined by visual examination with a dissection microscope. The binary individual survivorship data were then used to estimate a thermal survivorship curve for each collection using a logistic regression. We then estimated thermal tolerance as LD50 or the temperature at which 50% of the individuals survived.

In addition to the temperature and salinity measurements made at the time of collection, we also estimated the thermal environment experienced by individuals during development, as this has been shown to strongly influence thermal tolerance in adult copepods (Pereira et al., 2017; Sasaki & Dam, 2019). However, like other copepods, *Acartia* species exhibit an exponential relationship between temperature and development time (Kleppel et al., 1996; Mauchline, 1998; Miller et al., 1977; Peterson, 2001). These dual effects of temperature on thermal tolerance and development time are important to take into account. To do so, we used a continuous temperature record from the adjacent Mumford Cove (Baumann, 2020) and an approach similar to that used by Hirche et al. (2019) to examine the effects of developmental temperature on body size. Development time equations have been empirically derived in Leandro et al. (2006) for *Acartia tonsa* and Durbin and Durbin (1992) for *Acartia hudsonica* (*D* = 5,490*(*T* + 1)^−2.05^; *D* = 1,288*(*T* + 2.37)^−1.4774^, respectively). For each collection, we calculated the mean temperature (*T*_mean_) for increasingly larger intervals of time preceding collection (*t* = 1,2,…*n* days). A development time (D) estimate was then generated by substituting *T*_mean_ into the development time equation. If the resulting development time estimate was greater than the number of days in the time interval (t), we increased the length of the time interval by one day and re‐estimated development time with the new mean temperature. This process continued until the development time matched the time interval (i.e., where D(*T*_mean_) ≅ *t*). This process is illustrated in Appendix S2. We then estimated several different parameters for this time range, including the mean temperature, the average daily temperature range, and the absolute range of temperatures. We also estimated day length for each collection date using the *geospheres* package in R. Finally, a safety margin was estimated as the difference between the temperature measured at the time of collection and that collection's LD50 value.

We used an ANOVA to examine differences in the thermal survivorship curves between collections (Survivorship ~ Species*Collection). We also used an ANOVA to examine factors affecting LD50, the metric of thermal tolerance (LD50 ~ Species*(day length + mean developmental temperature + mean daily temperature range during development + absolute range of temperatures during development)). Finally, separate linear regressions were used to examine just the relationship between thermal tolerance and estimated mean developmental temperature for the two species.

To examine the adaptive mechanisms underlying observed differences between seasonal collections, we collected *Acartia tonsa* individuals several times during Summer and Fall 2019 to establish laboratory cultures. These cultures were maintained in common garden conditions for three generations to minimize the effects of previous environmental acclimation. For these collections, both mature females and males were sorted into filtered seawater, which was then slowly brought to 18°C. We chose this temperature because it represents an approximate mean temperature experienced by *Acartia tonsa* during its growth season, and because *Acartia tonsa* individuals from a wide range of thermal environments have been shown to survive and reproduce at this temperature (Sasaki & Dam, 2019). Cultures were kept at ambient CO_2_ concentrations. All cultures were maintained under a 12:12 light:dark cycle and fed *ad libitum* a mixture of a green flagellate (*Tetraselmis* sp.), a small diatom (*Thalassiosira weissflogii*), and a cryptomonad (*Rhodomonas salina*). Phytoplankton were cultured semi‐continuously in F/2 medium (without silica for *Tetraselmis* and *Rhodomonas*) under the same light cycle and temperature as the copepod cultures.

To establish experimental cultures, mature F2 females were isolated for several days and the eggs produced were collected and split into two groups. These groups developed at either 18°C or 24°C. We chose 24°C as the warm development temperature as this is commonly experienced during the summer in Long Island Sound. This allows us to compare not only the effects of genetic differentiation between seasonal collections, but also to examine any potential changes in the strength of developmental phenotypic plasticity occurring over the course of the year. Mature F3 females were exposed to a 24‐hr acute heat stress using the same protocol as the field individuals, with stress temperatures ranging between 25°C and 37°C.

Curves were again estimated using a logistic regression of individual survivorship against stress temperature, and thermal tolerance calculated as LD50. The difference in LD50 between the 18°C and 24°C developmental temperature groups, or the change in thermal tolerance as a result of an increase in developmental temperature (herein referred to as ΔLD50), represents the effects of developmental phenotypic plasticity. Standard error values for ΔLD50 were calculated as √(SE_18_^2^ + SE_24_^2^), where SE_18_ and SE_24_ are the standard error estimates for LD50 from the 18°C and 24°C developmental temperature groups, respectively. Differences between the logistic regressions for the seasonal collections were examined using an ANOVA (Survivorship ~ Stress temperature*Developmental temperature*Collection). Using the same reverse estimation approach as was used for the field individuals, we determined the environment experienced by the F0 individuals for each collection. We then tested the correlation between the strength of plasticity and the range of temperatures experienced by the F0 individuals. We also examined the correlation between thermal tolerance and the strength of developmental phenotypic plasticity.

Body size at maturity of F3 individuals from both developmental temperature groups was also measured. Approximately 60 individuals (30 females and 30 males) were collected and photographed using a camera attached to an inverted microscope. Body lengths were measured as the prosome length using ImageJ. Differences in body size between the various groups were examined using an ANOVA (Body size ~ Developmental temperature*Sex*Collection).

## 3 RESULTS

### 3.1 Experiments with Field Individuals

**Figure 1:**
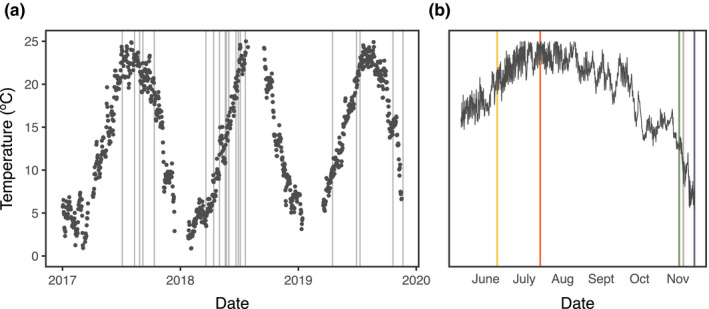
Water temperatures recorded near the site of collections. (a) Mean daily temperatures from 1 January 2017 to the final collection day, 17 November 2019. Collections involving unacclimated copepods (either *A. hudsonica* or *A. tonsa*) are indicated by the gray bars. (b) Water temperatures from June to November 2019, recorded at 30‐min intervals. Collections used in the A. tonsa common garden experiments are indicated by the colored bars

**Figure 2:**
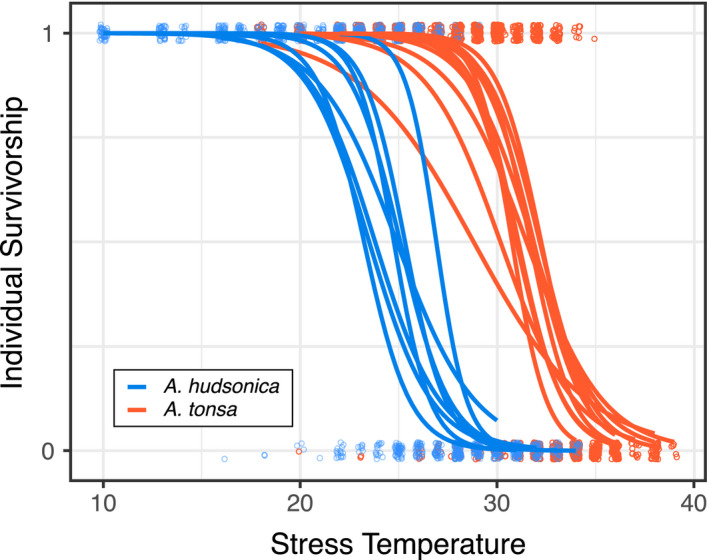
Thermal survivorship curves for unacclimated field‐collected individuals. Individual survivorship measurements are shown with points, 1 indicates the individual survived while mortality is indicated by 0. Survivorship curves for each collection were estimated using a logistic regression. Curves for the two species are shown in different colors

**Figure 3:**
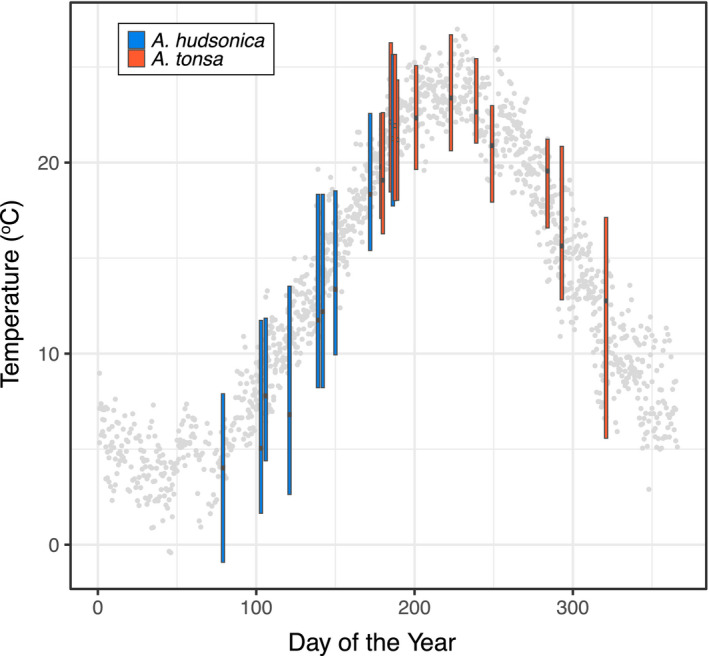
Range of developmental temperatures for each collection. Each collection is summarized in a single bar. Center points show the estimated mean developmental temperature, while top and bottom edges show maximum and minimum temperatures experienced during development, respectively. The two different species are shown in different colors. Gray points show the three years of mean daily temperature data covering the sampling period

Mean daily temperature ranged from <5°C in the winter to around 25°C during the summer (Figure 1). A total of 21 thermal survivorship curves were generated with 2760 nonacclimated field individuals during this time period, 12 curves for *Acartia tonsa* and 9 for *Acartia hudsonica* (Figure 2), with collections spanning nearly the entire range of temperatures observed in Long Island Sound (Appendix S1; Figure 1a). Copepods for the common garden experiments were collected at five times, also covering a large portion of the annual temperature range (Figure 1b). Measured water temperatures closely match those recorded in the continuous temperature record from the adjacent Mumford Cove (*r* = 0.98; *p* < 10^–14^). Based on the estimated developmental temperature regimes, both species experience larger degrees of variation over their season of occurrence than within individual generations (Figure 3).

**Figure 4:**
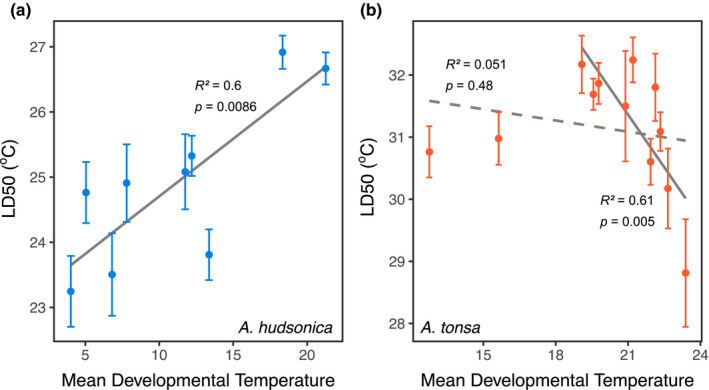
Thermal tolerance, measured as LD50 or the temperature of 50% mortality, plotted against the estimated mean developmental temperature for the two species. Error bars show standard error. Note the differences in both *x*‐ and *y*‐axes. Two regression lines are shown for *Acartia tonsa*, one for all collections (dashed line) and one for all collections except the two from Fall 2019 (solid line)

**Figure 5:**
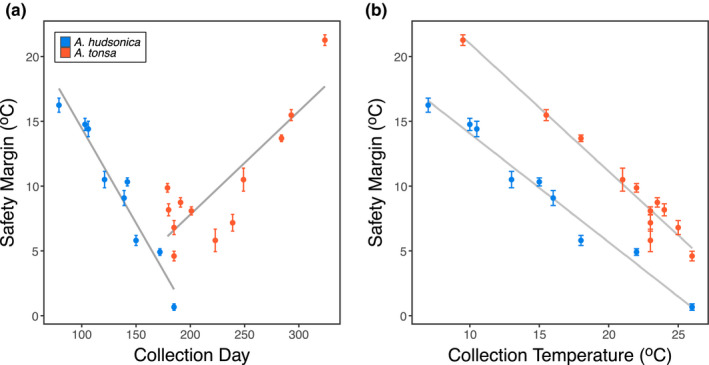
Thermal safety margins for field‐collected *Acartia* copepods calculated as the difference between thermal tolerance (LD50) and the measured water temperature at the time of collection. Thermal safety margins are plotted against (a) the day of the year copepods were collected and (b) the measured water temperature at the time of collection. The two different species are shown in different colors. Error bars show standard error

There were significant differences both between the thermal survivorship curves of the two species (*p* = <0.00001) and between collections (*p* = .026) within species (Figure 2; Appendix S3). The ANOVA for thermal tolerance values (LD50) suggests a marginally significant effect of mean developmental temperature (*p* = .096; Appendix S4), but significant differences between the two species (*p* = <10^–5^) and a significant mean developmental temperature × species interaction (*p* = .008). The linear regressions for thermal tolerance against mean developmental temperature were significant in both species, but only when two of the collections from Fall 2019 were excluded from the *A. tonsa* data set (Figure 4). These two collections occurred late in the season of occurrence and might indicate a seasonally dependent, nonlinear relationship between thermal tolerance and temperature; it would not be surprising if the influence of other environmental factors (pH, food abundance, etc.) changes the relationship between thermal tolerance and developmental temperature over the course of the annual temperature cycle. The regressions were in opposite directions for the two species; thermal tolerance was positively related to mean developmental temperature for *A. hudsonica*, but negatively related in *A. tonsa*. Both species had large thermal safety margins, exceeding 15°C at times (Figure 5a). Margins decreased as water temperatures increased for both species (Figure 5b). While *A. tonsa* maintained thermal safety margins of at least 5°C, safety margins in *A. hudsonica* approached 0°C during the warmest collections.

### 3.2 Common garden experiments

**Figure 6:**
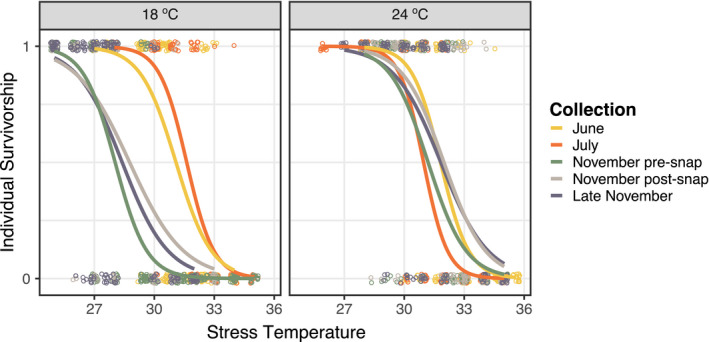
Thermal survivorship curves for F3 individuals from the *Acartia tonsa* split‐brood common garden experiment. Individual survivorship measurements are shown with points, 1 indicates the individual survived while mortality is indicated by 0. Survivorship curves for each collection were estimated using a logistic regression. Curves for the various seasonal collections are shown in different colors

**Figure 7:**
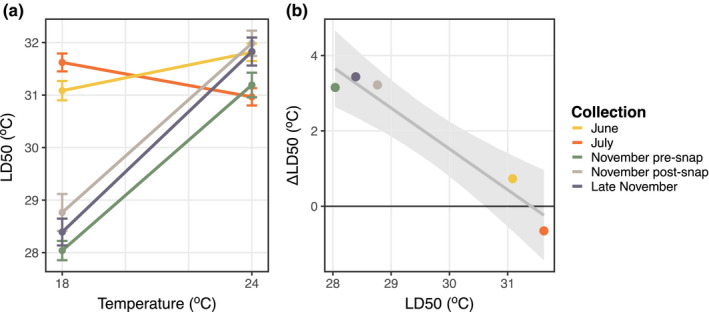
(a) Thermal tolerance reaction norms for the seasonal collections of *Acartia tonsa*, shown in different colors. Copepods were maintained under common garden conditions for several generations and then split into two groups which developed at either 18°C or 24°C. Error bars show standard error. (b) The correlation between thermal tolerance and the strength of phenotypic plasticity

After three generations of common garden conditions, there were still significant differences between the thermal survivorship curves for the various collections (*p* < 10^–5^; Figure 6; Appendix S5), indicating genetic differences between populations collected at different times of year. There was also a drastic difference in the strength of developmental phenotypic plasticity between collections (Figure 7a). This was also reflected in a significant interaction term between collection and developmental temperature in the ANOVA results (*p* < 10–^5^; Appendix S5). There was no correlation between temperature range experienced by the F0 generation and the strength of phenotypic plasticity (*r* = 0.60, *p* = .281), but there was a significant negative correlation between thermal tolerance and the strength of developmental phenotypic plasticity (*r* = −0.97, *p* < .01; Figure 7b); the collections from warmer months had higher thermal tolerances, but exhibited weaker phenotypic plasticity than those from colder collections.

**Figure 8:**
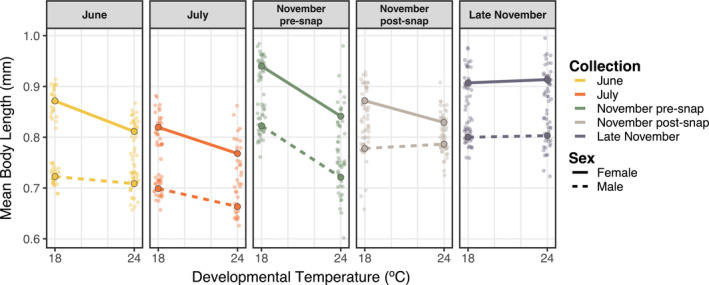
Body size reaction norms for F3 common garden *Acartia tonsa* individuals developed at either 18°C or 24°C. The mean body size for each group is shown as a large point, with the individual measurements shown as faint points behind it. The various collections are shown in different colors. The two sexes are shown with different line types

There were significant differences in body size between collections, sexes, and developmental temperatures (Appendix S6). Generally, copepods collected during warmer months were smaller than those from cooler months, female copepods were larger than male copepods, and body size decreased in the warmer developmental temperature (Figure 8). The strength of plasticity in body size also varied between collections, with collections from warm months and the presnap collection in November exhibiting more plasticity than the two from final collections, which exhibited either strongly decreased or no significant plasticity. There was no correlation between plasticity in body size and plasticity in thermal tolerance (*r* = 0.19, *p* = .764).

## 4 DISCUSSION

Temporal variation is an intrinsic property of the natural environment, with seasonality in temperature being one of the most pronounced forms of variation observed. We find that this strong seasonality produces intra‐annual variation in thermal tolerance in two species of short‐lived *Acartia* copepods from Long Island Sound. Common garden experiments show that the seasonal variation in *A. tonsa* is likely driven by genetic differentiation of both thermal tolerance and the strength of phenotypic plasticity. Body size and body size plasticity also vary significantly over the course of year in this species.

Developmental environments can play a large role in determining adult thermal tolerance in copepods (Healy et al., 2019; Pereira et al., 2017; Sasaki & Dam, 2019; Sasaki et al., 2019). Reverse estimating the developmental temperature experienced by copepods in the field revealed a significant effect of mean temperature on thermal tolerance. This effect, however, differed between the two species. *Acartia hudsonica* showed increased thermal tolerance as mean developmental temperatures increased, whereas *A. tonsa* showed decreasing thermal tolerance values as developmental mean temperatures increased. This may indicate that other factors affect thermal tolerance in the field; pH, for example, is strongly correlated with seasonal patterns of temperature in Long Island Sound (Baumann et al., 2015). Low pH has been shown to interact with increased temperature to decrease thermal tolerance (Paganini et al., 2014). The pH in the sampling area regularly drops below 7.6 during time periods when the water is warmest (Baumann, 2020), which may explain the reduction of thermal tolerance at higher developmental temperatures in unacclimated *A. tonsa* individuals. This highlights the critical nature of interactions between multiple environmental and ecological factors in the determination of thermal limits in natural populations. Day length is also known to play an important role as an environmental cue for aspects of copepod physiology involved in diapause (Baumgartner & Tarrant, 2017; Hairston & Kearns, 1995). As a highly reliable indicator of seasonality, it may also play a role in the determination of thermal tolerance. The lack of a strong effect in our results, however, suggests that day length is likely not the main determinant of the observed seasonal changes in thermal tolerance.

Despite the unexpected relationship between developmental temperature and thermal tolerance, *A. tonsa* always maintained a positive thermal safety margin, suggesting that realized selection on upper thermal limits may be weak. By contrast, summer temperatures in Long Island Sound already approach maximum thermal tolerance levels of *A. hudsonica*, and future warming may decrease the window of seasonal occurrence for this species. Conversely, warming may increase the time period *Acartia tonsa* occurs in Long Island Sound as the lower limit of their distribution is currently determined by the production of resting eggs at low temperatures rather than a lower lethal thermal limit (Sullivan & McManus, 1986). Studies in nearby systems have suggested that temperature plays an important role in the seasonal switch between *A. hudsonica* and *A. tonsa* dominated plankton communities, driven primarily by effects on egg production (Sullivan & McManus, 1986). Our results suggest that differences in the upper thermal limits between the two species may also play an important role in determining seasonal occurrence.

There is clear evidence for abundant genetic variation in both thermal tolerance and phenotypic plasticity within this population of *Acartia tonsa*, as drastic differences were observed between collections during common garden experiments. The large population sizes, short generation times (on the order of weeks), obligate sexual reproductive mode, and presence of overlapping generations characteristic of many copepod species in temperate regions are expected to promote the maintenance of genetic variation by fluctuating selection (Ellner & Hairston, 1994; Ellner & Sasaki, 1996; Svardal et al., 2014). The large magnitude, predictable seasonal variation in temperature is also expected to select for the maintenance of phenotypic plasticity, as opposed to other strategies, like bet‐hedging, which are selected for by unpredictable variation (Liu et al., 2019; Simons, 2009). However, theory predicts that plasticity should inhibit balanced polymorphism (Crispo, 2008; Ellner & Hairston, 1994). Our observations of both strong phenotypic plasticity and genetic polymorphism are contrary to this prediction. The negative relationship observed between thermal tolerance and the strength of phenotypic plasticity may be an important but overlooked promoter of the maintenance of balanced polymorphism in short‐lived organisms. This negative relationship is commonly viewed as a reflection of a trade‐off between plasticity and thermal tolerance (Stillman, 2003), but there are several other mechanisms or processes that would generate this relationship (van Heerwaarden & Kellermann, 2020). Interestingly, this relationship also appears to shape patterns of plasticity in *A. tonsa* across large spatial scales (Sasaki & Dam, 2019).

The reaction norms generated in the common garden experiments do, however, indicate that there may be some cost associated with plasticity. When raised at higher temperatures, the November collections were able to match, or even exceed the thermal tolerance values of the June and July copepods. If there was no cost associated with plasticity, we would expect this population of *Acartia tonsa* to be able to respond to seasonal variation in temperature purely though the effects of developmental phenotypic plasticity. Instead, we see a decrease in plasticity during the warmest times, possibly indicating some advantage of specialist genotypes over more plastic genotypes (Gilchrist, 1995). One potential explanation is that the maintenance of plasticity exacts some energetic cost (DeWitt et al., 1998) and is selected against during the warmest months when phytoplankton abundance is lowest in these environments (Lopez et al., 2014).

Coincident with the seasonal fluctuations in selection on thermal tolerance are changes in selection on reproduction. In multivoltine copepods like *Acartia*, there is an expected trade‐off between reproductive efficiency and reproductive output across the seasonal temperature cycle (Omori, 1997). At high temperatures, selection favors small body sizes that maximize reproductive efficiency (the ratio between egg production and respiration), while lower temperatures favor larger body sizes and increased output. Seasonal variation in plasticity of body size has been overlooked as an important component of this dynamic in life‐history adaptation. We observed the strongest plasticity in body size during the warmest times of the year when selection is for small females and efficient egg production. This likely represents adaptive plasticity, as a temperature‐driven decrease in body size may increase the fitness of the female during the warmest times of the year. Similarly, the reduced plasticity in body size observed in some of the November collections may help copepods take advantage of seasonal pulses in food availability; reduced plasticity during winter would prevent a reduction in body size and reproductive output when water temperatures begin to increase during the onset of the spring phytoplankton bloom.

Adaptive responses to relatively short timescale environmental variation have impacts on long‐term evolutionary dynamics (Bell, 2010). Deciphering observed patterns of adaptation across seasonal timescales is crucial for our ability to predict population responses to climate change, especially as fluctuating selection may promote the maintenance of adaptive genetic variation in a population (Stern & Lee, 2020). In Long Island Sound, as in many temperate regions, the most prominent warming has been observed during winter (Dahlke & Maturilli, 2017; Preston, 2004; Record et al., 2019; Rice & Stewart, 2016; Wu et al., 2017). We observed strong phenotypic plasticity in thermal tolerance in the late fall, but not during the summer. As phenotypic plasticity allows for a rapid increase in thermal tolerance, this may reduce vulnerability to the most immediate effects of climate change. However, strong phenotypic plasticity may also dampen selection and prevent long‐term adaptation to warming (Crispo, 2008). By contrast, the lack of observed plasticity in summer copepods may make the population more susceptible to increases in temperature, but promote genetic adaptation by increasing the efficacy of selection. The seasonal variation in both thermal tolerance and phenotypic plasticity suggests abundant genetic variation for both traits, which may allow for rapid responses to environmental changes.

Previous work has uncovered several deeply diverged cryptic lineages in *Acartia tonsa*, distributed across the Northwest Atlantic (Caudill & Bucklin, 2004; Chen & Hare, 2011; Sasaki & Dam, 2019). While it is possible that the observed seasonal variation in thermal adaptation results from fluctuations in the relative abundance of sympatric cryptic lineages, it is unlikely that this seasonal variation represents the effects of gene flow or immigration of individuals from adjacent sites; past work has shown no variation in thermal tolerance or plasticity in *Acartia tonsa* collected from sites ranging from the Gulf of Mexico to the Bay of Fundy (Sasaki & Dam, 2019). Further, clades that do appear to be differentially adapted are strongly structured by salinity (Plough et al., 2018; Sasaki & Dam, 2019) and could not persist at our sampling site in Long Island Sound, which has only minor salinity fluctuations throughout the year.

It is unclear which adaptive mechanisms produce the variable thermal tolerance values observed for *Acartia hudsonica* in this study. It is possible that this species also adapts to fluctuating conditions through a combination of phenotypic plasticity and genetic differentiation, but common garden experiments are required to identify the underlying mechanisms. However, there are environmental differences between the seasons of occurrence between this species and *Acartia tonsa* that may affect the patterns of adaptation. Most notably, water temperatures near our collection site are less variable during winter than during summer (Appendix S7). If plasticity evolves in response to variability in the environment, there may be weaker selection for plasticity in *A. hudsonica* than in *A. tonsa*, and this species might instead rely more on genetic polymorphism of stress tolerance. Alternatively, if patterns in plasticity are dictated by energetic costs, we might expect the response of *A. hudsonica* to seasonal variation to be accomplished entirely by phenotypic plasticity, as phytoplankton abundance is generally higher during their season of occurrence. Understanding the relative contributions of genetic polymorphism and phenotypic plasticity to adaptation to seasonal variation is a key component in our understanding of how populations will respond to climate change and requires integrative approaches.

## CONFLICT OF INTEREST

None declared.

## AUTHOR CONTRIBUTION

**Matthew Sasaki:** Conceptualization (equal); Data curation (equal); Formal analysis (equal); Funding acquisition (equal); Investigation (equal); Methodology (equal); Visualization (equal); Writing‐original draft (equal); Writing‐review & editing (equal). **Hans Dam:** Conceptualization (equal); Funding acquisition (equal); Methodology (equal); Writing‐original draft (equal); Writing‐review & editing (equal).

## Supporting information

Appendix S1‐S7

## Data Availability

All data and R scripts used in analyses are available as an R Project folder on Dryad. Citation: Sasaki, M.C. & H.G. Dam. 2021. Genetic differentiation underlies seasonal variation in thermal tolerance, body size, and plasticity in a short‐lived copepod, Dryad Dataset, https://doi.org/10.5061/dryad.9kd51c5dg
