# Supplementary material for: Genetic differentiation underlies seasonal variation in thermal tolerance, body size, and plasticity in a short‐lived copepod
Source: Ecol Evol. 2020 Oct 5;10(21):12200–10. doi: 10.1002/ece3.6851 (PMC7663071; doi:10.1002/ece3.6851)

**Appendix**

Appendix S1 – Collection details for each sampling event included in this study.

| **Date** | **Component** | **Species** | **Collection Temperature** |
| --- | --- | --- | --- |
| 7/5/17 | Field TPCs | *Acartia tonsa* | 25 |
| 8/12/17 | Field TPCs | *Acartia tonsa* | 23 |
| 8/28/17 | Field TPCs | *Acartia tonsa* | 23 |
| 9/7/17 | Field TPCs | *Acartia tonsa* | 21 |
| 10/12/17 | Field TPCs | *Acartia tonsa* | 18 |
| 3/21/18 | Field TPCs | *Acartia hudsonica* | 7 |
| 4/14/18 | Field TPCs | *Acartia hudsonica* | 10 |
| 5/2/18 | Field TPCs | *Acartia hudsonica* | 13 |
| 5/20/18 | Field TPCs | *Acartia hudsonica* | 16 |
| 5/23/18 | Field TPCs | *Acartia hudsonica* | 15 |
| 5/31/18 | Field TPCs | *Acartia hudsonica* | 18 |
| 6/22/18 | Field TPCs | *Acartia hudsonica* | 22 |
| 6/29/18 | Field TPCs | *Acartia tonsa* | 22 |
| 7/5/18 | Field TPCs | Both | 26 |
| 7/21/18 | Field TPCs | *Acartia tonsa* | 23 |
| 4/17/19 | Field TPCs | *Acartia Hudsonica* | 10.5 |
| 6/28/19 | Common Garden | *Acartia tonsa* | 23 |
| 6/30/19 | Field TPCs | *Acartia tonsa* | 24 |
| 7/11/19 | Field TPCs | *Acartia tonsa* | 23.5 |
| 7/29/19 | Common Garden | *Acartia tonsa* | 23 |
| 10/21/19 | Field TPCs | *Acartia tonsa* | 15.5 |
| 11/6/19 | Common Garden | *Acartia tonsa* | 13.5 |
| 11/9/19 | Common Garden | *Acartia tonsa* | 8 |
| 11/17/19 | Field TPCs & Common garden | *Acartia tonsa* | 9.5 |

Appendix S2 – A schematic representation of how developmental time period was reverse estimated from the continuous temperature record. The mean temperature was calculated for a period of time of *t* days. This mean temperature *T_mean_* was then used to estimate development time, *D*, using species-specific empirically estimated development time equations. If the estimated development time was larger than the time interval used, the time interval was increased by one day, and repeated. If the estimated development time matched the time interval used, that time interval was determined to be the development time.

Appendix S3 – ANOVA results comparing thermal survivorship curves between collections of unacclimated individuals from the field.

|  | **Df** | **Deviance** | **Resid. Df** | **Resid. Dev** | **Pr(>Chi)** |
| --- | --- | --- | --- | --- | --- |
| Stress Temperature | 1 | 1083.13 | 2379 | 2160.2 | < 2.2e-16 |
| Species | 1 | 634.87 | 2378 | 1525.4 | < 2.2e-16 |
| Collection | 1 | 4.96 | 2377 | 1520.4 | 0.02599 |
| Stress Temp. x Species | 1 | 0.12 | 2376 | 1520.3 | 0.72989 |
| Stress Temp. x Collection | 1 | 35.45 | 2375 | 1484.8 | 2.62E-09 |
| Species x Collection | 1 | 7.5 | 2374 | 1477.3 | 0.00616 |

Appendix S4 - ANOVA results comparing effects of estimated developmental environmental variables on thermal tolerance (estimated as LD50, or the temperature inducing 50% mortality), between collections of unacclimated individuals from the field.

|  | **Df** | **Sum Sq** | **Mean Sq** | **F value** | **Pr(>F)** |
| --- | --- | --- | --- | --- | --- |
| Species | 1 | 198.543 | 198.543 | 277.3586 | 3.77E-09 |
| Day Length | 1 | 2.111 | 2.111 | 2.9484 | 0.113949 |
| Mean Temperature | 1 | 2.374 | 2.374 | 3.3166 | 0.095868 |
| Mean Daily Temp. Range | 1 | 0.352 | 0.352 | 0.4911 | 0.497984 |
| Absolute Temp. Range | 1 | 0.043 | 0.043 | 0.0606 | 0.81007 |
| Species x Day Length | 1 | 1.936 | 1.936 | 2.7041 | 0.128338 |
| Species x Mean Temperature | 1 | 7.484 | 7.484 | 10.4546 | 0.007968 |
| Species x Mean Daily Temp. Range | 1 | 1.077 | 1.077 | 1.5039 | 0.245677 |
| Species x Absolute Temp. Range | 1 | 0.073 | 0.073 | 0.1025 | 0.75487 |
| Residuals | 11 | 7.874 | 0.716 |  |  |

Appendix S5 – ANOVA results comparing the thermal survivorship curves between the collections from the common garden experiments.

|  | **Df** | **Deviance** | **Resid. Df** | **Resid. Dev** | **Pr(>Chi)** |
| --- | --- | --- | --- | --- | --- |
| Stress Temperature | 1 | 717.19 | 1627 | 1541 | < 2.2e-16 |
| Collection | 4 | 81.22 | 1623 | 1459.8 | < 2.2e-16 |
| Developmental Temperature | 1 | 120.16 | 1622 | 1339.6 | < 2.2e-16 |
| Stress Temp. x Collection | 4 | 68.8 | 1618 | 1270.8 | 4.07E-14 |
| Stress Temp. x Dev. Temp. | 1 | 15.39 | 1617 | 1255.4 | 8.73E-05 |
| Collection x Dev. Temp | 4 | 80.02 | 1613 | 1175.4 | < 2.2e-16 |
| Stress Temp. x Collection x Dev. Temp | 4 | 3.11 | 1609 | 1172.3 | 0.5402 |

Appendix S6 - ANOVA results comparing the body size measurements between collections from the common garden experiments.

|  | **Df** | **Sum Sq** | **Mean Sq** | **F value** | **Pr(>F)** |
| --- | --- | --- | --- | --- | --- |
| Collection | 4 | 0.91615 | 0.22904 | 210.755 | < 2.2e-16 |
| Sex | 1 | 1.68949 | 1.68949 | 1554.624 | < 2.2e-16 |
| Developmental Temperature | 1 | 0.21191 | 0.21191 | 194.995 | < 2.2e-16 |
| Collection x Sex | 4 | 0.06354 | 0.01588 | 14.616 | 2.38E-11 |
| Collection x Dev. Temp. | 4 | 0.17489 | 0.04372 | 40.232 | < 2.2e-16 |
| Sex x Dev. Temp. | 1 | 0.01642 | 0.01642 | 15.11 | 0.0001139 |
| Collection x Sex x Dev. Temp. | 4 | 0.01973 | 0.00493 | 4.539 | 0.0012945 |
| Residuals | 547 | 0.59445 | 0.00109 |  |  |

Appendix S7 - Daily temperature ranges from Mumford Cove plotted against the day of the year. A Loess smoother was used to visualize the annual trend in temperature range.


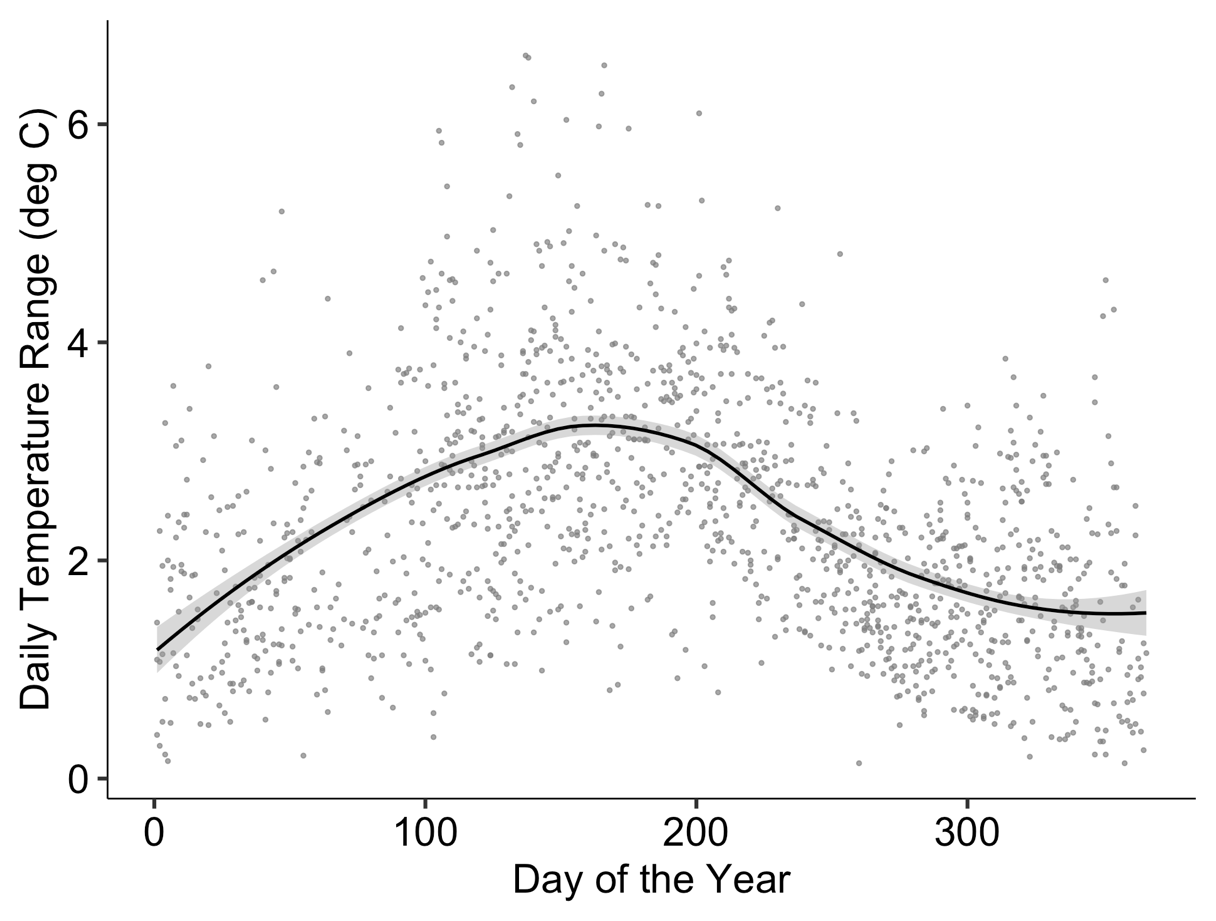

Supplement: Supplementary file 1 — Appendix S1‐S7 [file ECE3-10-12200-s001.docx]
